# Meta-analysis of predictors of healthcare-associated *Clostridioides difficile* infection

**DOI:** 10.1017/ash.2024.413

**Published:** 2024-11-14

**Authors:** Jesse Fajnzylber, William M. Patterson, Neil Nero, Adrian V. Hernandez, Abhishek Deshpande

**Affiliations:** 1 Cleveland Clinic Lerner College of Medicine of Case Western Reserve University, Cleveland, OH, USA; 2 Education Institute, Floyd D. Loop Alumni Library, Cleveland Clinic, Cleveland, OH, USA; 3 Health Outcomes, Policy, and Evidence Synthesis (HOPES) Group, University of Connecticut School of Pharmacy, Storrs, CT, USA; 4 Unidad de Revisiones Sistemáticas y Meta-análisis (URSIGET), Vicerrectorado de Investigación, Universidad San Ignacio de Loyola (USIL), Lima, Peru; 5 Center for Value-Based Care Research, Primary Care Institute, Cleveland Clinic, Cleveland, OH, USA; 6 Department of Infectious Diseases, Respiratory Institute, Cleveland Clinic, Cleveland, OH, USA

## Abstract

Our systematic review and meta-analysis of 40 studies (*n* = 3,905,559) identified gastric acid suppressants, recent hospitalization, antibiotic exposure, and certain comorbidities as independent predictors of healthcare-associated *Clostridioides difficile* infection (HA-CDI) among adult inpatients. Targeted antibiotic stewardship and judicious use of gastric acid suppressants can reduce the incidence of HA-CDI.

## Introduction

*Clostridioides difficile* infection (CDI) affects >500,000 people annually in the United States.^1^ Older age, hospitalization, and exposure to high-risk antibiotics are known risk factors for healthcare-associated CDI (HA-CDI);^2^ however, literature on some risk factors are conflicting. Gastric acid suppressants (GASs), particularly proton pump inhibitors (PPIs), have been associated with CDI, but some studies have suggested that this association may represent confounding rather than an intrinsic effect of PPIs.^3^ A deeper understanding of current and new risk factors for HA-CDI can help clinicians and healthcare epidemiologists triage effective preventive measures. The objective of this study was to systematically evaluate and update the most common predictors for developing HA-CDI in adult inpatients.

## Methods

The study adhered to the Transparent Reporting of multivariable prediction models for Individual Prognosis Or Diagnosis: checklist for Systematic Reviews and Meta-Analyses (TRIPOD-SMRA) guidelines.^4^ The checklist and protocol are available in the supplement.

### Eligibility criteria

We conducted searches of Medline, EMBASE, and the Cochrane Library from database inception until July 25, 2023. The complete search strategy is available in the supplement. Two investigators (JF and WP) screened abstracts of any design or language against prespecified inclusion criteria: (i) admitted for ≥48 hr before CDI diagnosis; (ii) adult inpatients (≥18 years); (iii) analysis used a multivariable analytic model and provided effect measures at the patient level; and (iv) HA-CDI was a study end point. For abstracts that met inclusion/exclusion criteria, the same methodology was applied to the full text. Discrepancies were resolved by a third researcher (AD).

### Study evaluation

Model data were independently extracted (JF and WP) using the CHecklist for critical Appraisal and the data extraction for systematic Reviews of prediction Modeling Studies (CHARMS) checklist and assessed for risk of bias (ROB) using the Quality In Prognosis Studies (QUIPS) tool.^5,6^ Additional details are included in the supplement.

### Statistical analyses

Risk factors were meta-analyzed if there were ≥3 estimates from ≥3 studies. An inverse variance random effects model was utilized to estimate effects of factors on CDI as odds ratios (ORs) with 95% confidence intervals (CIs). Heterogeneity was quantified with the *I*^2^ metric. Publication bias was assessed with funnel plots and Egger’s test. Where asymmetry was detected, publication bias was assessed using the nonparametric trim-and-fill method.

## Results

**Figure 1.**
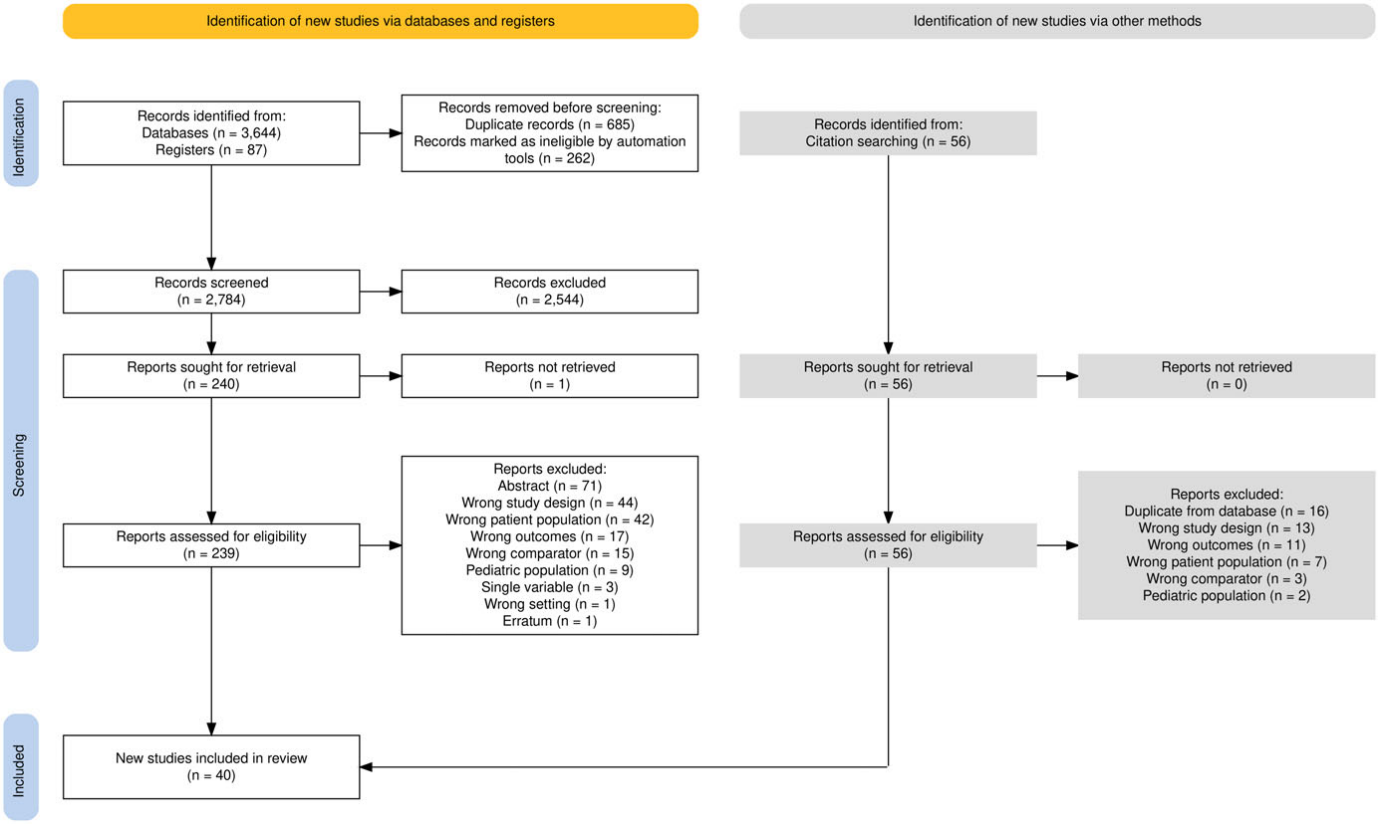
PRISMA flow diagram for study selection.

We found 3,731 records matching search criteria, of which 2,784 were deemed eligible after deduplication. After abstract and full-text review, 40 studies (n = 3,905,559) met eligibility criteria and are included in the supplemental references (Figure 1).

ROB varied across studies, with 15% (n = 6), 58% (n = 23), and 28% (n = 11) having low, moderate, and high ROB, respectively (Table S1, Figure S1). The domain most frequently identified as high ROB was “bias due to confounding” (Domain 5, high risk of bias [13%, n = 5]). “Bias in statistical analysis and reporting” (Domain 6) had the fewest ratings of low ROB (48%, n = 19, Figure S1).

Of 321 risk factors identified across 40 studies, 23 had three or more independent estimates and were eligible for meta-analysis. The most common risk factors (by number of estimates) were use of GAS (n = 20), PPI use (n = 13), hospitalization in the last 2–3 months (n = 9), antibiotic use (n = 8), age in years (n = 7), and sex (n = 7). An overview of all risk factors are in the supplement.

### Gastric acid suppressants

**Figure 2.**
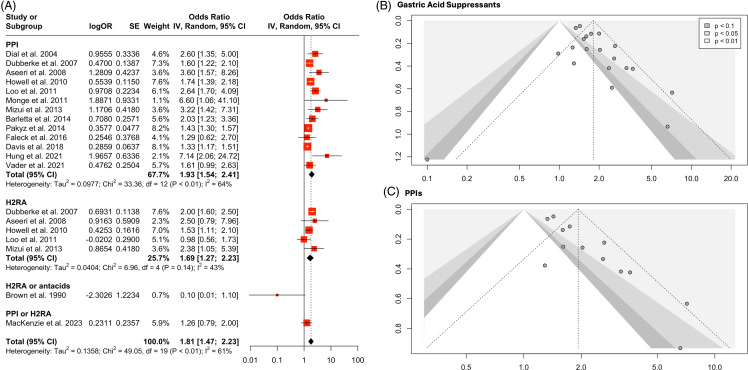
(A) Forest plot for all gastric acid suppressants. Funnel plots for (B) any type of gastric acid suppressant and (C) PPIs alone. Each estimate is plotted as a point, with estimate precision on the *y*-axis against the magnitude of the study’s measured effect. Shading indicates various thresholds for statistical significance, with the unshaded region representing p-values greater than 0.1.

Meta-analysis of 20 point estimates from 15 studies (n = 326,240) demonstrated that receipt of GAS was a significant predictor of HA-CDI (OR 1.81, 95%CI: 1.47–2.23, *I*^2^ = 61%, Figure 2A). A total of 13 studies evaluated PPIs (n = 307,283) and five evaluated H2RAs (n = 153,586) compared to no acid suppression. Meta-analysis demonstrated significantly higher odds of HA-CDI with PPIs (OR 1.93, 95%CI: 1.54–2.41, *I*^2^ =64%) and H2RAs (OR 1.69, 95%CI: 1.27-2.23, *I*^2^ =43%). Of the 13 studies analyzing PPIs, six also adjusted for comorbidities, and four for age. In a subgroup analysis of studies adjusting for both (n =3), the pooled effect size was OR 1.73 (95%CI: 0.94–3.18, *I*^2^ =71%, Figure S2). Funnel plots appear asymmetric for both GAS and PPIs (Figure 2B-C**)**, further confirmed by Egger’s test in GAS (intercept = 1.12, 95%CI: 0.20–2.04 P = 0.03) and PPIs (intercept = 1.94, 95%CI: 1.18–2.69, *P* <.001). Adjustment for publication bias resulted in a GAS OR of 1.54 (95%CI: 1.18–2.02) with five studies trim-and-filled and a PPI OR of 1.50 (95%CI: 1.07–2.09) with six studies trim-and-filled. H2RAs had <10 estimates, thus ineligible for publication bias analyses.

Results of the other risk factors are included in the supplement (Table S2, Figures S3–S6).

## Discussion

The goal of this meta-analysis was to both better establish the predictors for developing HA-CDI and meta-analyze the risk estimates for hospitalized adults. Consistent with previous reports, increased age, systemic antibiotic use, previous hospitalization, and presence of certain comorbidities were significant predictors of HA-CDI.^1^ Use of GAS (H2RA and PPIs) increased the odds of developing HA-CDI by 70%–90%.

The relationship between GAS and CDI remains controversial, as some studies have found a significant association, whereas others have not.^1,7–9^ Our findings suggest that the receipt of any GAS is a significant predictor of HA-CDI; however, these results should be interpreted with caution due to publication bias and substantial heterogeneity among the studies. In a subgroup analysis of studies adjusting for age and comorbidities, the PPI effect size remained largely unchanged, suggesting that confounding does not fully account for the observed relationship. Though guidelines from the Infectious Diseases Society of America (IDSA) state there is insufficient evidence to recommend discontinuing PPIs purely as a preventive measure against CDI, they advocate for stewardship activities aimed at discontinuing unnecessary PPI use.^10^ All inpatients on chronic GAS should have their medications reviewed on admission and discontinued if unnecessary.

In our study, we meta-analyzed predictors of HA-CDI when ≥3 studies provided adjusted measures. Due to space constraints, our discussion is focused on GAS. However, we also found that receipt of antibiotics, recent hospitalization, advanced age, hypoalbuminemia, comorbidities such as solid tumor malignancies, congestive heart failure, and renal failure, certain therapies such as dialysis and mechanical ventilation were associated with significantly increased risk of HA-CDI.

Our study has several limitations. Though many potential risk factors were present across multiple studies, some were discretized differently, vaguely worded, or used a different reference level. All assumptions made to combine variables are reported in our supplement. Second, our results reflect the ROB present across included studies. Authors often did not adjust for previously described risk factors (eg, age, antibiotics, comorbidity burden) that we decided *a priori* must be included. More than half of the included studies had a “moderate” ROB, most frequently introduced in the statistical analysis and reporting domain. Lastly, incidence of CDI varied between studies which is reflected by different risk factor point estimates resulting in moderate–high heterogeneity.

In conclusion, our meta-analysis offers further evidence and contributes to a better understanding of the most important predictors for HA-CDI. This study highlights the multifactorial nature of HA-CDI, identifying the most significant predictors of HA-CDI. However, not all risk factors are modifiable. Infection control and stewardship programs would greatly benefit by focusing on the judicious use of antibiotics and GAS, as these are the most easily modifiable interventions.

## Supporting information

Fajnzylber et al. supplementary material 1 Fajnzylber et al. supplementary material

Fajnzylber et al. supplementary material 2 Fajnzylber et al. supplementary material

Fajnzylber et al. supplementary material 3 Fajnzylber et al. supplementary material

Fajnzylber et al. supplementary material 4 Fajnzylber et al. supplementary material

Fajnzylber et al. supplementary material 5 Fajnzylber et al. supplementary material

Fajnzylber et al. supplementary material 6 Fajnzylber et al. supplementary material

Fajnzylber et al. supplementary material 7 Fajnzylber et al. supplementary material

Fajnzylber et al. supplementary material 8 Fajnzylber et al. supplementary material

## Data Availability

Data collection forms for risk of bias and model data extraction are available in the supplement. Extracted data and associated code are available from the authors upon reasonable request.

## Supplementary material

The supplementary material for this article can be found at https://doi.org/10.1017/ash.2024.413

## Registration and protocol

This review was not registered. The review protocol is included in the supplement.
