# Supplementary figures and images for: Meta-analysis of predictors of healthcare-associated Clostridioides difficile infection
Source: Antimicrob Steward Healthc Epidemiol. 2024 Nov 14;4(1):e202. doi: 10.1017/ash.2024.413 (PMC11574607; doi:10.1017/ash.2024.413)

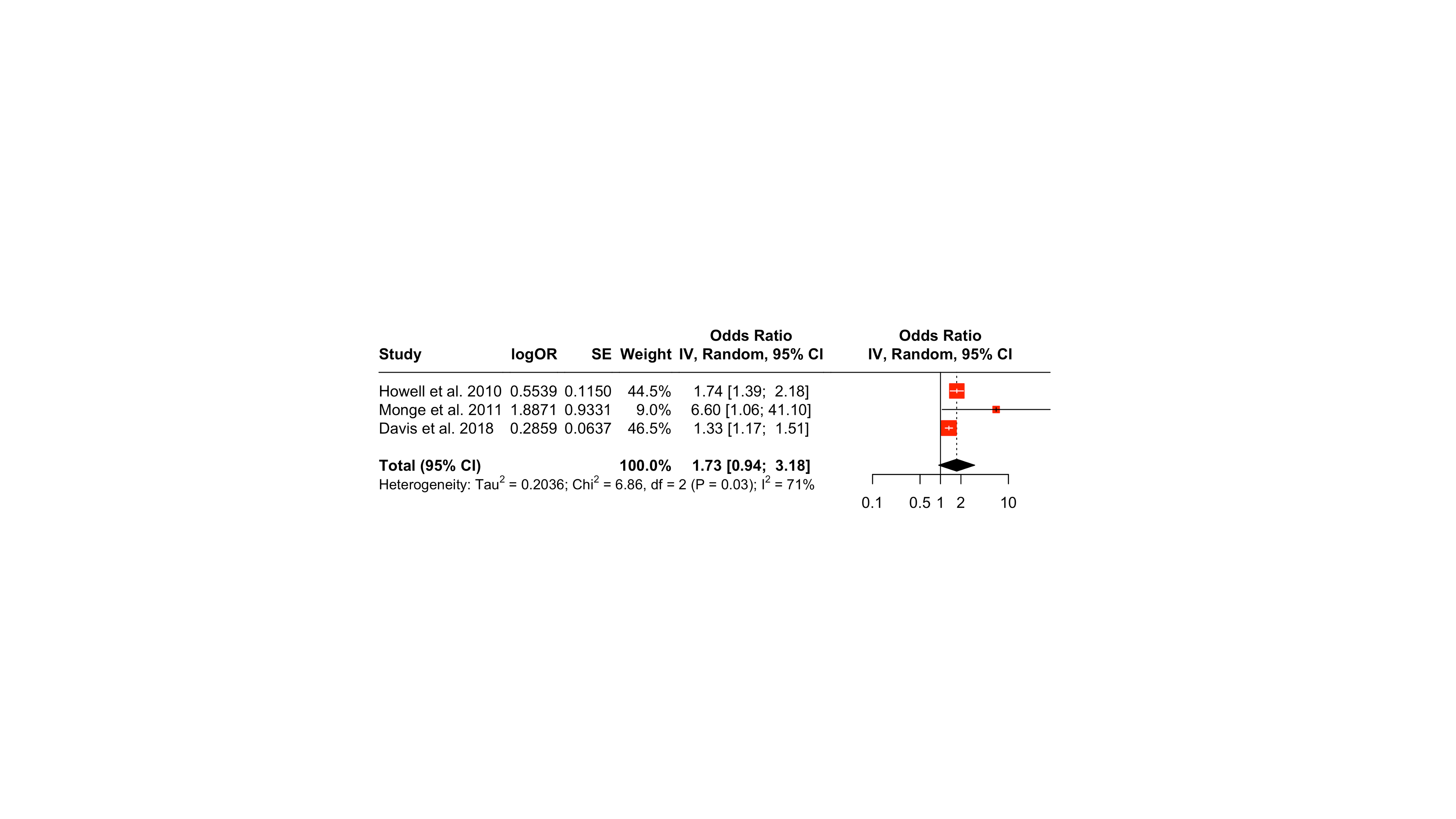

Supplement: Fajnzylber et al. supplementary material 2 — Fajnzylber et al. supplementary material [file S2732494X24004133sup002.tiff]

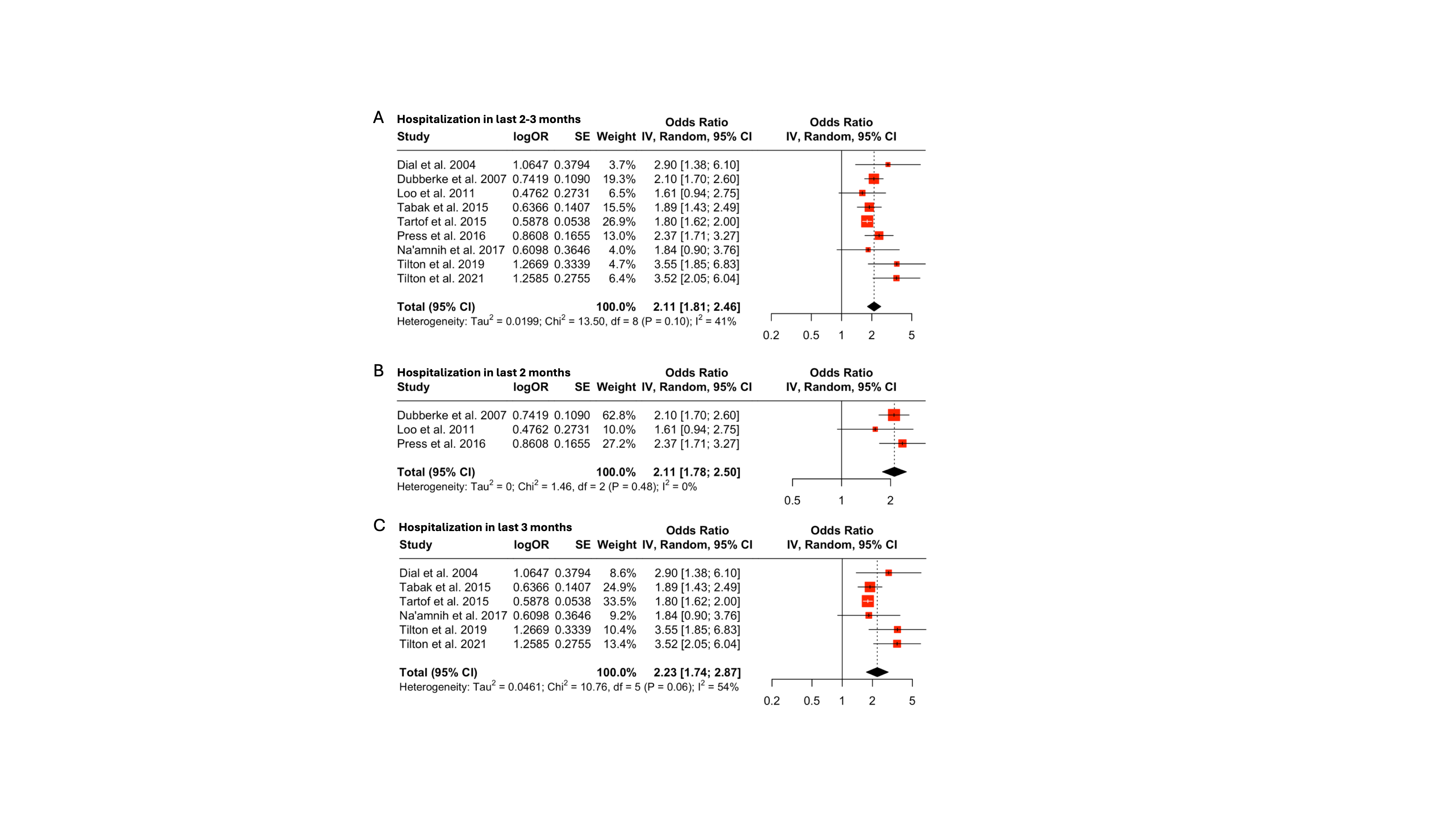

Supplement: Fajnzylber et al. supplementary material 3 — Fajnzylber et al. supplementary material [file S2732494X24004133sup003.tiff]

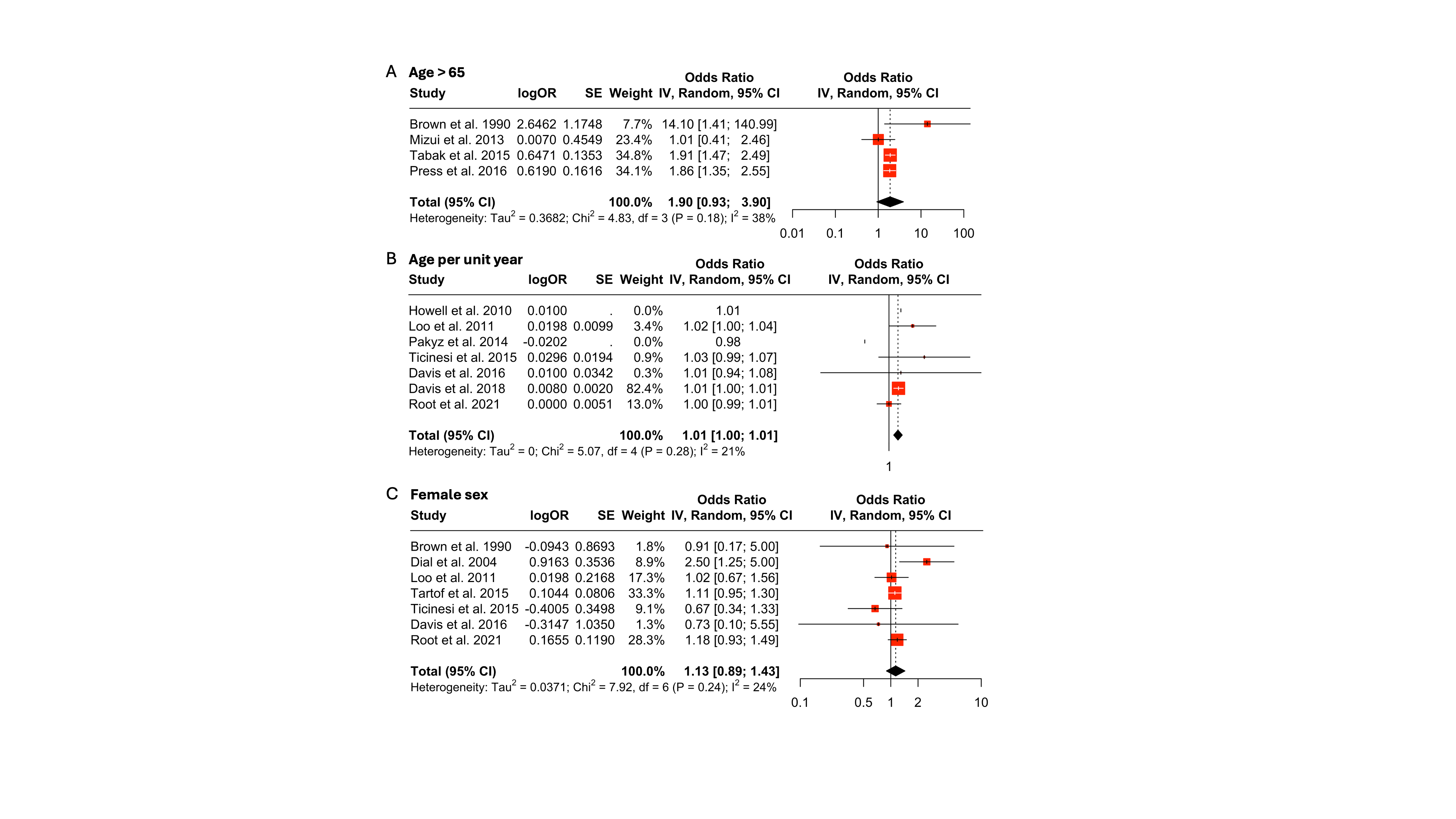

Supplement: Fajnzylber et al. supplementary material 4 — Fajnzylber et al. supplementary material [file S2732494X24004133sup004.tiff]

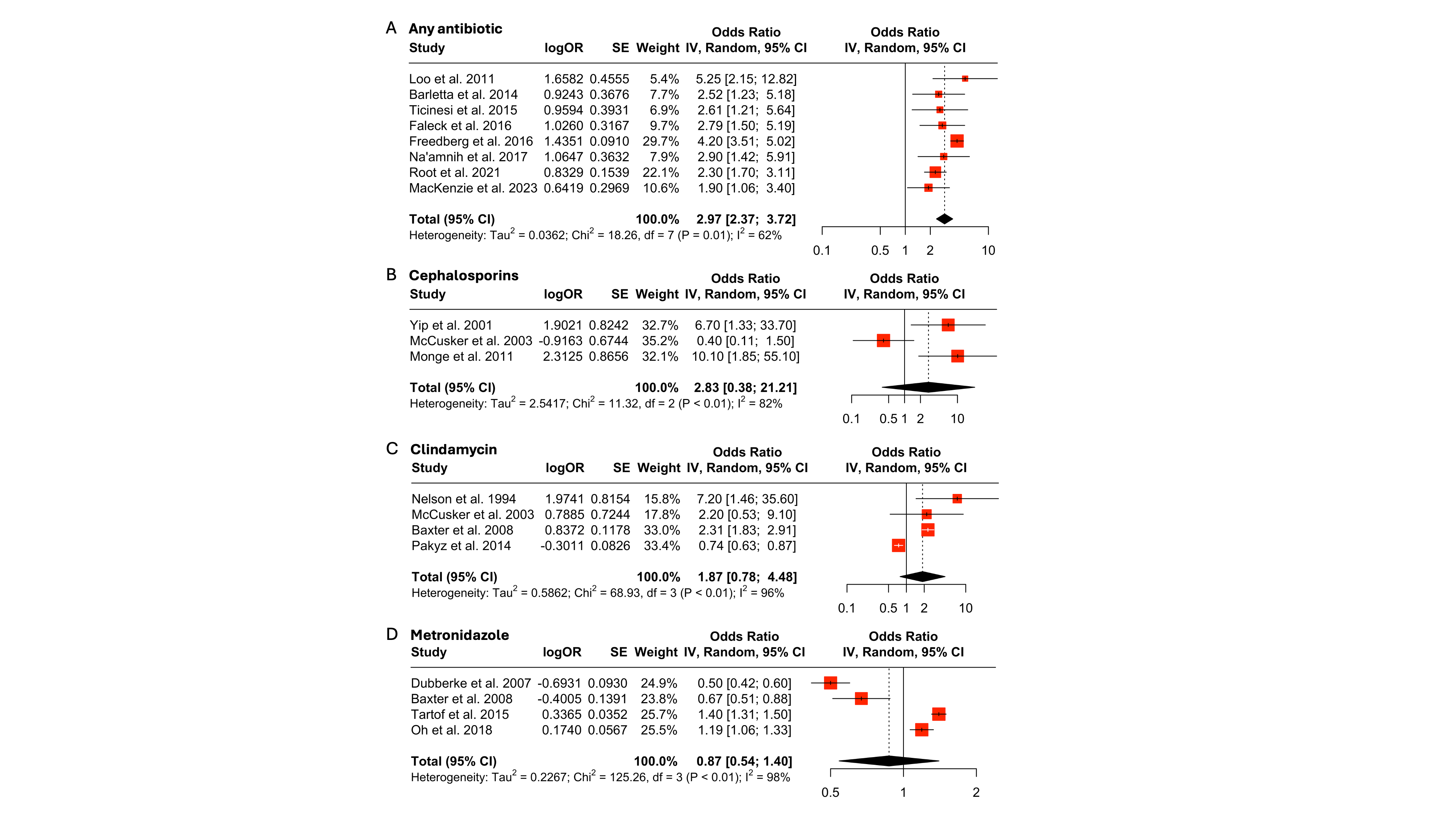

Supplement: Fajnzylber et al. supplementary material 5 — Fajnzylber et al. supplementary material [file S2732494X24004133sup005.tiff]

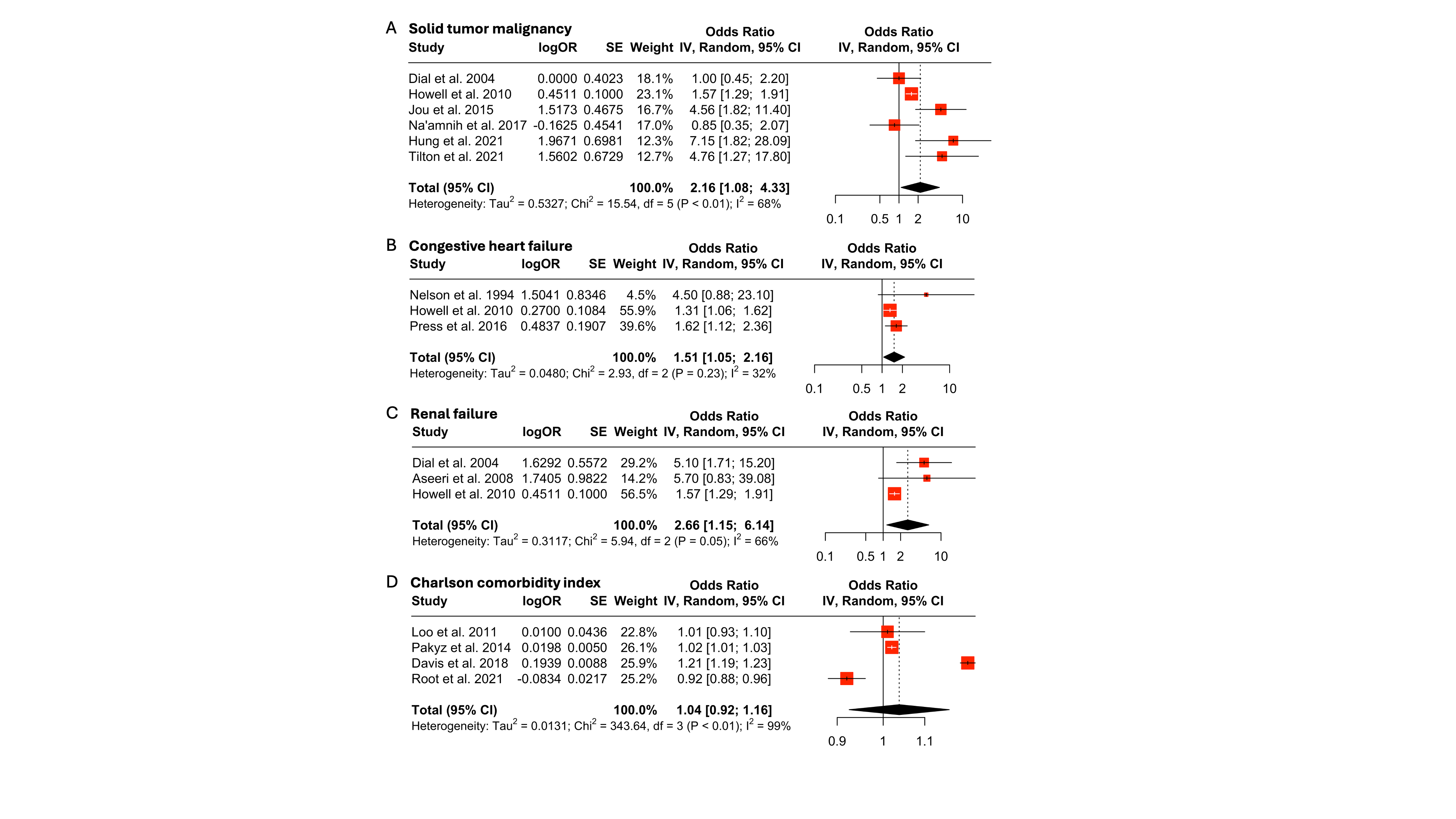

Supplement: Fajnzylber et al. supplementary material 6 — Fajnzylber et al. supplementary material [file S2732494X24004133sup006.tiff]
