# Supplementary material for: Meta-analysis of predictors of healthcare-associated Clostridioides difficile infection
Source: Antimicrob Steward Healthc Epidemiol. 2024 Nov 14;4(1):e202. doi: 10.1017/ash.2024.413 (PMC11574607; doi:10.1017/ash.2024.413)
Supplement: Fajnzylber et al. supplementary material 7 — Fajnzylber et al. supplementary material [file S2732494X24004133sup007.docx]

**Supplemental Material**

**Methods**

**Protocol:**

CHARMs: Extracted data in the CHARMs checklist included: sources, countries, study populations, participant types (e.g., ICU, medicine ward), outcome to be predicted, candidate predictors, number of events, sample sizes, missing data, model development, performance, evaluation, data presentation, interpretation, and discussion, point estimates, and model adjustments.

**Assumptions for combining factors:**

**Age:** Many studies reported age as a continuous variable, which allows us to estimate the marginal effect of an increase of 1 year on HOCDI risk. Other studies categorized age with a variety of thresholds: either data-driven or defined a priori. For studies that explicitly used 65 as a threshold (i.e., >65 years vs. ≤65 years, *n* = 4), we were able to estimate an effect. For studies that used data-driven thresholds, or for those that chose non-standard reference groups, we were unable to combine results across studies.

**Antibiotic use (generic):** Many studies reported an effect of generic antibiotic use (yes/no). Since it has been generally established that some, but not all, antibiotics can increase the risk for HOCDI, the clinical utility of an effect estimate is limited. Further, some studies only looked at antibiotic use during hospitalization, whereas others included antibiotic use preceding hospitalization. Regardless, we combined only factors for which papers included an explicit lookback time for data collection.

**Cancer:** We chose to combine only coefficients that estimate a risk for *solid* tumor malignancies that were active during the observed hospitalization (e.g., a history of cancer was not sufficient). Though the risk conferred by liquid tumors was of interest, we did not have sufficient estimates to meta-analyze this factor. We chose not to combine these groups based on clinical judgment that the risk from these two groups is likely not comparable.

**Antibiotic use (specific classes and duration):** A number of studies reported estimates for specific classes or generations of antibiotics, stratified antibiotic use by the duration of treatment with a specific class, or presented a unique grouping of antibiotic classes. While of great clinical interest, we did not have a sufficient number of estimates to meta-analyze different drug classes or specific durations of treatment.

**Gastric acid suppression:** We combined only estimates for those who received a medication indicated for acid suppression (e.g. PPI, H2RA) vs. those who did not. We included estimates from samples that included patients who started their PPI prior to hospitalization because PPIs address a symptom of an underlying pathology which may contribute to CDI risk.

Congestive heart failure: We combined estimates for ‘chronic heart failure’ and ‘congestive heart failure,’ as we assumed these refer to the same underlying pathology.

**Dialysis:** We combined four estimates of risk for dialysis. Three of these sampled patients without regard for dialysis indication, while one included only patients who had end-stage renal disease as their dialysis indication.

Sex: Studies use the terms sex and gender interchangeably, but we assumed all studies referred to biological sex as a risk factor. We chose male as our reference level and inverted estimates that used female.

**Recent hospitalization:** When defining a lookback period for recent hospitalizations, studies used a variety of thresholds (e.g. 3 months, 90 days, last year) and contrasts (e.g., vs. no admission ever, vs. no admission in last year). We assumed that estimates of risk for individuals with a hospitalization in the preceding 3 months (including samples that had a lookback period of any timeframe shorter than 3 months) could be reasonably combined. We also performed a subgroup analysis of estimates for a lookback period of 2 months (or 60 days) vs. 3 months (or 90 days).

**Hypoalbuminemia:** Pathology laboratories define limits of normal based upon the population they serve, which is reflected in the variety of cutpoints for hypoalbuminemia in the literature. We chose to combine estimates for explicitly labeled hypoalbuminemia and albumin levels that were described as hypoalbuminemic in the text of the paper.

**Complete search strategy:**

A comprehensive search strategy was developed with a trained medical librarian (N.N.) using the following terms: (*Clostridioides* OR *Clostridium* OR *C difficile*) AND (predict* OR risk*) AND (model* OR tool*). Indexing terms and keywords were combined with Boolean and proximity operators. No search limits were applied, and an additional 56 records were identified based on manual review of the literature and papers cited in recent systematic reviews of HA-CDI.^1,2^

- **Search Strategies for all databases queried**
- **Ovid MEDLINE(R) ALL <1946 to July 24, 2023>**
- 1 Clostridioides difficile/ 11365
- 2 (clostridioides difficile or clostridium difficile or txid1496 or c diff or cdiff or c difficile or bacillus difficilis or clostridium difficilis or peptoclostridium difficile).mp. 19631
- 3 1 or 2 19631
- 4 ((predict* or risk*) and (model* or tool*)).mp. 1429343
- 5 3 and 4 1076
- **Embase <1974 to 2023 July 24>**
- 1 clostridioides difficile/ 4745
- 2 (clostridioides difficile or clostridium difficile or txid1496 or c diff or cdiff or c difficile or bacillus difficilis or clostridium difficilis or peptoclostridium difficile).mp. 39611
- 3 1 or 2 39611
- 4 ((predict* or risk*) and (model* or tool*)).mp. 1895353
- 5 3 and 4 2315
- **Cochrane Database of Systematic Reviews & Cochrane Central Register of Controlled Trials**
- **July 2023 (87 results)**
- #1 MeSH descriptor: [Clostridioides difficile] this term only 318
- #2 (("clostridioides difficile" or "clostridium difficile" or "txid1496" or "c diff" or "cdiff" or "c NEXT difficile" or "bacillus difficilis" or "clostridium difficilis" or "peptoclostridium difficile")):ti,ab,kw 1602
- #3 #1 or #2 1602
- #4 ((predict* or risk*) and (model* or tool*)):ti,ab,kw 79599
- #5 #3 and #4 87

**Article screening:**

The article selection process occurred in two rounds using the Covidence systematic review software (Veritas Health Innovation, Melbourne, Australia). Our pre-specified criteria excluded studies that did not differentiate between CA-CDI and HA-CDI, or which only reported univariate analyses. Additionally, we excluded studies where both reviewers determined the findings were not generalizable to a hospitalized patient. For this reason, studies in which the entire control group had diarrhea; that analyzed a disease-specific cohort (e.g., patients with cirrhosis); or looked only at hospital- or community-level factors were excluded in this analysis. Records with abstract-only content (conference proceedings) were also excluded, as we lacked sufficient detail to evaluate study design and analytic methodologies.

**Model extraction and risk of bias:**

The CHARMS checklist contains two sections that help reviewers systematically identify key elements of study design (e.g., inclusion/exclusion criteria, search strategies) and specific reporting elements that are essential for evaluating multivariable regression analyses.

ROB is a qualitative measure assigned to a specific domain of a study (e.g., outcome assessment, study confounding) that represents the likelihood that the predictive accuracy of the model may be biased by that domain. QUIPS provides a standardized tool to assess ROB. Each domain has three to seven specific questions and prompts raters for an overall judgment of ROB based on responses to these questions. Each rater makes objective and subjective judgements of validity for each domain based on the summation of the issues, then judges the overall ROB as low, moderate, or high. Certain domain prompts require *a priori* key characteristics (**Table S3**). Inter-rater differences in domain-level ROB were discussed and agreed upon between reviewers. If at least one domain was rated as high risk or more than three were moderate risk, the overall study risk was considered high. If all domains were rated as low risk, the overall study risk was considered low. All other combinations were considered moderate risk.

**Statistical analyses:**

Several studies had ≥1 point estimate for the same risk factor variable; for example, those that reported univariable analyses or reported ≥1 multivariable regression model. In such cases we used the model that adjusted for the most clinically relevant confounders. The authors decided *a priori* the most likely contributors to confounding would be exposure to antibiotics, age, and a combined measure of the burden of comorbidities (e.g., Charlson comorbidity index). For non-numeric variables, we additionally required the reference group to remain the same. This method recalculates the effect size given the presence of publication bias.^3^ Statistical analyses were carried out with R 4.3.1 using the *meta* package.^4,5^ Heterogeneity was defined using the *I^2^* metric, where <30% is low, 30-60% is moderate, >60% is substantial.

**Results**

The majority of studies (n = 30, 75%) were conducted in the US (**Table S3**), with Canada (n = 4, 10%) as the next most frequent.^6–45^ Most studies were single-center (IQR: 1 - 3.5), with a median sample size of 4,493 (IQR: 472 - 79,202). The median number of HA-CDI events was 178 (IQR: 67.8 - 598). Most studies (n = 35, 88%) were retrospective; all were observational. Most studies used a cohort (n = 24, 60%) or case-control (n = 17, 43%) design; only one study (3%) reported a cross-sectional design. Two papers presented results of hybrid study designs: one was a nested case-control^21^ and the other performed an independent case-control study.^14^

**Recent hospitalization**

Nine studies (n = 610,643) estimated the effect of hospitalization in the 2-3 months prior to the index admission on the risk of HA-CDI. Meta-analysis of the studies demonstrated that any hospitalization during that time period was a significant predictor of HA-CDI (OR: 2.11, 95% CI: 1.81 - 2.46, *I*^2^ = 41%). Lookback times were also analyzed separately. Three studies (n = 128,714) were meta-analyzed for hospitalization in the last two months, with a pooled OR of HA-CDI of 2.11 (95% CI: 1.78 – 2.50, *I*^2^ = 0%). Six studies (n = 481,929) were combined for hospitalization in the last three months, with a pooled OR of 2.23 (95% CI: 1.74 – 2.87, *I*^2^ = 54%; **Figure S3**).

**Exposure to antibiotics**

Eight studies (n = 190,435) adjusted for the effect of antibiotics either during or three months preceding the index hospitalization and meta-analysis of the studies showed that exposure to antibiotics was a significant predictor of HA-CDI. Compared to those who did not receive antibiotics during this time, those who did had nearly three times increased odds of contracting HA-CDI (OR: 2.97, 95% CI: 2.37 – 3.72, *I*^2^ = 62%, **Figure S4A**). Only three antibiotic classes had enough estimates for conducting a meta-analysis, none of which were significantly associated with HA-CDI. Three studies (n = 222) were meta-analyzed for cephalosporins (OR: 2.83, 95% CI: 0.38 – 21.21, *I*^2^ = 82%), four studies (n = 18,816) for clindamycin (OR: 1.87, 95% CI: 0.78 – 4.48, *I*^2^ = 96%) and four studies (n = 815821) for metronidazole (OR; 0.87, 95% CI: 0.54 – 1.40, *I*^2^ = 98%, **Figure S4B-D**).

**Demographics**

Meta-analysis of seven studies (n = 243,282) reported that age was a significant predictor of HA-CDI, and each additional year increased the odds (OR = 1.01, 95% CI: 1.00-1.01, *I*^2^ = 21%, **Figure S5A**). Four studies (n = 161,690) dichotomized age at 65, with those older having a non-significant OR of 1.90 (95% CI: 0.93- 3.90, *I*^2^ = 38%, **Figure S5B**). Seven studies (n = 432,811) evaluated the effect of sex (female vs. male) and reported that sex was not a significant predictor of HA-CDI. Compared to males, females had an OR of 1.13 (95% CI: 0.89, 1.43, *I*^2^= 24%, **Figure S5C**).

**Comorbidities**

Many individual comorbidities were significant predictors of HA-CDI. Six studies (n = 105,155) adjusted for solid tumor malignancies, more than doubling the odds of HA-CDI (OR = 2.16, 95% CI: 1.08 – 4.33, *I*^2^ = 68%). Three studies were meta-analyzed for both congestive heart failure (OR = 1.51, 95% CI: 1.05 – 2.16, *I*^2^ = 32%; n = 182,219) and renal failure (OR = 2.66, 95% CI: 1.15 - 6.14, *I*^2^ = 66%; n = 103,265) and were significantly associated with developing HA-CDI. Four studies (n = 140,853) were meta-analyzed for Charlson comorbidity index, which was not significantly associated with HA-CDI (OR = 1.04, 95% CI: 0.92 - 1.16, *I*^2^ = 99%, **Figure S6**).

**Supplemental References**

1. Puro N, Joseph R, Zengul FD, Cochran KJ, Camins BC, Ray M. Predictors of Hospital-Acquired Clostridioides difficile Infection: A Systematic Review. *J Healthc Qual Off Publ Natl Assoc Healthc Qual*. 2020;42(3):127-135.

2. Rao K, Dubberke ER. Can prediction scores be used to identify patients at risk of Clostridioides difficile infection? *Curr Opin Gastroenterol*. 2022;38(1):7-14.

3. Duval S, Tweedie R. Trim and fill: A simple funnel-plot-based method of testing and adjusting for publication bias in meta-analysis. *Biometrics*. 2000;56(2):455-463.

4. Viechtbauer W. Conducting Meta-Analyses in R with the metafor Package. *J Stat Softw*. 2010;36:1-48.

5. R Core Team. *R: A Language and Environment for Statistical Computing*. R Foundation for Statistical Computing; 2023.

6. Ahyow LC, Lambert PC, Jenkins DR, Neal KR, Tobin M. Bed occupancy rates and hospital-acquired Clostridium difficile infection: a cohort study. *Infect Control Hosp Epidemiol*. 2013;34(10):1062-1069.

7. Aseeri M, Schroeder T, Kramer J, Zackula R. Gastric acid suppression by proton pump inhibitors as a risk factor for clostridium difficile-associated diarrhea in hospitalized patients. *Am J Gastroenterol*. 2008;103(9):2308-2313.

8. Barletta JF, Sclar DA. Proton pump inhibitors increase the risk for hospital-acquired Clostridium difficile infection in critically ill patients. *Crit Care Lond Engl*. 2014;18(6):714.

9. Baxter R, Ray GT, Fireman BH. Case-Control Study of Antibiotic Use and Subsequent Clostridium difficile–Associated Diarrhea in Hospitalized Patients. *Infect Control Hosp Epidemiol*. 2008;29(1):44-50.

10. Brown E, Talbot GH, Axelrod P, Provencher M, Hoegg C. Risk factors for Clostridium difficile toxin-associated diarrhea. *Infect Control Hosp Epidemiol*. 1990;11(6):283-290.

11. Brown KA, Fisman DN, Moineddin R, Daneman N. The Magnitude and Duration of Clostridium difficile Infection Risk Associated with Antibiotic Therapy: A Hospital Cohort Study. *PLoS ONE*. 2014;9(8):e105454.

12. Davis BM, Yin J, Blomberg D, Fung ICH. Impact of a prevention bundle on Clostridium difficile infection rates in a hospital in the Southeastern United States. *Am J Infect Control*. 2016;44(12):1729-1731.

13. Davis ML, Sparrow HG, Ikwuagwu JO, Musick WL, Garey KW, Perez KK. Multicentre derivation and validation of a simple predictive index for healthcare-associated Clostridium difficile infection. *Clin Microbiol Infect Off Publ Eur Soc Clin Microbiol Infect Dis*. 2018;24(11):1190-1194.

14. Dial S, Alrasadi K, Manoukian C, Huang A, Menzies D. Risk of Clostridium difficile diarrhea among hospital inpatients prescribed proton pump inhibitors: cohort and case-control studies. *CMAJ Can Med Assoc J J Assoc Medicale Can*. 2004;171(1):33-38.

15. Dubberke ER, Reske KA, Yan Y, Olsen MA, McDonald LC, Fraser VJ. Clostridium difficile--associated disease in a setting of endemicity: identification of novel risk factors. *Clin Infect Dis Off Publ Infect Dis Soc Am*. 2007;45(12):1543-1549.

16. Eddi R, Malik MN, Shakov R, Baddoura WJ, Chandran C, Debari VA. Chronic kidney disease as a risk factor for Clostridium difficile infection. *Nephrol Carlton Vic*. 2010;15(4):471-475.

17. Faleck DM, Salmasian H, Furuya EY, Larson EL, Abrams JA, Freedberg DE. Proton Pump Inhibitors Do Not Increase Risk for Clostridium difficile Infection in the Intensive Care Unit. *Am J Gastroenterol*. 2016;111(11):1641-1648.

18. Forster AJ, Daneman N, van Walraven C. Influence of antibiotics and case exposure on hospital-acquired Clostridium difficile infection independent of illness severity. *J Hosp Infect*. 2017;95(4):400-409.

19. Freedberg DE, Salmasian H, Cohen B, Abrams JA, Larson EL. Receipt of Antibiotics in Hospitalized Patients and Risk for Clostridium difficile Infection in Subsequent Patients Who Occupy the Same Bed. *JAMA Intern Med*. 2016;176(12):1801-1808.

20. Garey KW, Dao-Tran TK, Jiang ZD, Price MP, Gentry LO, Dupont HL. A clinical risk index for Clostridium difficile infection in hospitalised patients receiving broad-spectrum antibiotics. *J Hosp Infect*. 2008;70(2):142-147.

21. Howell MD, Novack V, Grgurich P, et al. Iatrogenic gastric acid suppression and the risk of nosocomial Clostridium difficile infection. *Arch Intern Med*. 2010;170(9):784-790.

22. Hung YP, Lee JC, Tsai BY, et al. Risk factors of Clostridium difficile-associated diarrhea in hospitalized adults: Vary by hospitalized duration. *J Microbiol Immunol Infect Wei Mian Yu Gan Ran Za Zhi*. 2021;54(2):276-283.

23. Jou J, Ebrahim J, Shofer FS, et al. Environmental transmission of Clostridium difficile: association between hospital room size and C. difficile Infection. *Infect Control Hosp Epidemiol*. 2015;36(5):564-568.

24. Loo VG, Bourgault AM, Poirier L, et al. Host and pathogen factors for Clostridium difficile infection and colonization. *N Engl J Med*. 2011;365(18):1693-1703.

25. MacKenzie EL, Murillo C, Bartlett AH, Marrs R, Landon EM, Ridgway JP. Clostridioides difficile colonization and the frequency of subsequent treatment for C. difficile infection in critically ill patients. *Infect Control Hosp Epidemiol*. 2023;44(11):1782-1787.

26. McCusker ME, Harris AD, Perencevich E, Roghmann MC. Fluoroquinolone Use and Clostridium difficile–Associated Diarrhea. *Emerg Infect Dis*. 2003;9(6):730-733.

27. McFarland LV, Surawicz CM, Stamm WE. Risk factors for Clostridium difficile carriage and C. difficile-associated diarrhea in a cohort of hospitalized patients. *J Infect Dis*. 1990;162(3):678-684.

28. Mizui T, Teramachi H, Tachi T, et al. Risk factors for Clostridium difficile-associated diarrhea and the effectiveness of prophylactic probiotic therapy. *Pharm*. 2013;68(8):706-710.

29. Monge D, Morosini M, Millán I, et al. [Risk factors for Clostridium difficile infections in hospitalized patients]. *Med Clin (Barc)*. 2011;137(13):575-580.

30. Mora AL, Salazar M, Pablo-Caeiro J, et al. Moderate to high use of opioid analgesics are associated with an increased risk of Clostridium difficile infection. *Am J Med Sci*. 2012;343(4):277-280.

31. Motzkus-Feagans CA, Pakyz A, Polk R, Gambassi G, Lapane KL. Statin use and the risk of Clostridium difficile in academic medical centres. *Gut*. 2012;61(11):1538-1542.

32. Na’amnih W, Adler A, Miller-Roll T, Cohen D, Carmeli Y. Incidence and Risk Factors for Community and Hospital Acquisition of Clostridium difficile Infection in the Tel Aviv Sourasky Medical Center. *Infect Control Hosp Epidemiol*. 2017;38(8):912-920.

33. Nelson DE, Auerbach SB, Baltch AL, et al. Epidemic Clostridium difficile-associated diarrhea: role of second- and third-generation cephalosporins. *Infect Control Hosp Epidemiol*. 1994;15(2):88-94.

34. Oh J, Makar M, Fusco C, et al. A Generalizable, Data-Driven Approach to Predict Daily Risk of Clostridium difficile Infection at Two Large Academic Health Centers. *Infect Control Hosp Epidemiol*. 2018;39(4):425-433.

35. Pakyz AL, Jawahar R, Wang Q, Harpe SE. Medication risk factors associated with healthcare-associated Clostridium difficile infection: a multilevel model case-control study among 64 US academic medical centres. *J Antimicrob Chemother*. 2014;69(4):1127-1131.

36. Press A, Ku BS, McCullagh L, Rosen L, Richardson S, McGinn T. Developing a Clinical Prediction Rule for First Hospital-Onset Clostridium difficile Infections: A Retrospective Observational Study. *Infect Control Hosp Epidemiol*. 2016;37(8):896-900.

37. Root ED, Lindstrom M, Xie A, Mangino JE, Moffatt-Bruce S, Hebert C. Investigating the association of room features with healthcare-facility-onset Clostridioides difficile: An exploratory study. *Infect Control Hosp Epidemiol*. 2021;42(7):847-852.

38. Tabak YP, Johannes RS, Sun X, Nunez CM, McDonald LC. Predicting the risk for hospital-onset Clostridium difficile infection (HO-CDI) at the time of inpatient admission: HO-CDI risk score. *Infect Control Hosp Epidemiol*. 2015;36(6):695-701.

39. Tartof SY, Rieg GK, Wei R, Tseng HF, Jacobsen SJ, Yu KC. A Comprehensive Assessment Across the Healthcare Continuum: Risk of Hospital-Associated Clostridium difficile Infection Due to Outpatient and Inpatient Antibiotic Exposure. *Infect Control Hosp Epidemiol*. 2015;36(12):1409-1416.

40. Ticinesi A, Nouvenne A, Folesani G, et al. Multimorbidity in elderly hospitalised patients and risk of Clostridium difficile infection: a retrospective study with the Cumulative Illness Rating Scale (CIRS). *BMJ Open*. 2015;5(10):e009316.

41. Tilton CS, Johnson SW. Development of a risk prediction model for hospital-onset Clostridium difficile infection in patients receiving systemic antibiotics. *Am J Infect Control*. 2019;47(3):280-284.

42. Tilton CS, Sexton ME, Johnson SW, et al. Evaluation of a risk assessment model to predict infection with healthcare facility-onset Clostridioides difficile. *Am J Health-Syst Pharm AJHP Off J Am Soc Health-Syst Pharm*. 2021;78(18):1681-1690.

43. Vader DT, Weldie C, Welles SL, Kutzler MA, Goldstein ND. Hospital-acquired Clostridioides difficile infection among patients at an urban safety-net hospital in Philadelphia: Demographics, neighborhood deprivation, and the transferability of national statistics. *Infect Control Hosp Epidemiol*. 2021;42(8):948-954.

44. Yip C, Loeb M, Salama S, Moss L, Olde J. Quinolone use as a risk factor for nosocomial Clostridium difficile-associated diarrhea. *Infect Control Hosp Epidemiol*. 2001;22(9):572-575.

45. Zilberberg MD, Tabak YP, Sievert DM, et al. Using electronic health information to risk-stratify rates of Clostridium difficile infection in US hospitals. *Infect Control Hosp Epidemiol*. 2011;32(7):649-655.

**Table S1:** General study information and risk of bias

| **Title** | **Study Location** | **Number of study centers** | **Study start year** | **Study end year** | **Sample size** | **Events** | **Age (mean or median)** | **Males (%)** | **Study design** | **Diagnostic test** | **Overall QUIPS Risk of Bias** |
| --- | --- | --- | --- | --- | --- | --- | --- | --- | --- | --- | --- |
| Brown et al. 1990 | United States | 1 | 1,987 | 1,987 | 74 | 37 | 63.00 | 50.0 | Retrospective case-control | Cytotoxin assay | High |
| McFarland et al. 1990 | United States | 1 | 1,985 | 1,986 | 728 | 31 |  |  | Prospective cohort | Cytotoxin assay | Moderate |
| Nelson et al. 1994 | United States | 1 | 1,988 | 1,999 | 99 | 33 |  |  | Retrospective case-control | CTA | Moderate |
| Yip et al. 2001 | United States | 1 | 1,998 | 1,998 | 81 | 27 | 73.00 | 48.0 | Retrospective case-control | EIA | Moderate |
| McCusker et al. 2003 | United States | 4 | 2,001 | 2,001 | 90 | 30 | 72.00 |  | Retrospective case-control | EIA | High |
| Dial et al. 2004 | Canada | 2 | 2,002 | 2,003 | 1,281 | 175 | 74.20 | 45.0 | Retrospective cohort and retrospective case-control | Cytotoxin assay (tissue culture) | Moderate |
| Dubberke et al. 2007 | United States | 1 | 2,003 | 2,003 | 36,086 | 382 |  | 42.0 | Retrospective cohort | EIA | Moderate |
| Aseeri et al. 2008 | United States | 1 | 2,005 | 2,006 | 188 | 94 |  | 43.6 | Retrospective case-control | EIA | High |
| Baxter et al. 2008 | United States | 16 | 1,999 | 2,005 | 4,493 | 1,142 | 68.00 | 48.0 | Retrospective case-control | EIA | Low |
| Garey et al. 2008 | United States | 1 | 2,005 | 2,007 | 54,226 | 392 | 58.00 | 44.6 | Retrospective cohort | Tissue culture cell cytotoxicity assay | Moderate |
| Eddi et al. 2010 | United States | 1 | 2,006 | 2,007 | 564 | 188 | 71.50 | 45.0 | Retrospective case-control | EIA | High |
| Howell et al. 2010 | United States | 1 | 2,004 | 2,008 | 101,796 | 665 | 65.40 | 41.0 | Retrospective cohort and retrospective case-control | Toxin assay | Moderate |
| Loo et al. 2011 | Canada | 6 | 2,006 | 2,007 | 12,304 | 117 |  | 50.0 | Prospective cohort | EIA | Low |
| Monge et al. 2011 | Spain | 1 | 2,006 | 2,006 | 51 | 38 | 73.00 | 55.3 | Retrospective case-control | NAAT / PCR, EIA | Moderate |
| Mora et al. 2011 | United States | 1 | 2,005 | 2,008 | 32,775 | 192 | 58.00 | 45.0 | Retrospective cohort | CTA | Moderate |
| Zilberberg et al. 2011 | United States | 85 | 2,007 | 2,008 | 2,022,213 | 4,963 |  |  | Retrospective cohort | Toxin assay | Moderate |
| Motzkus-Feagans et al. 2012 | United States | Unspecified subset of 107 hospitals | 2,002 | 2,009 | 109,568 | 31,472 |  | 51.2 | Retrospective case-control | Any inpatient with ICD-9 008.45 started on metronidazole or vancomycin PO for ≥3 days after ≥5 days of admission | Moderate |
| Ahyow et al. 2013 | United Kingdom | 3 | 2,006 | 2,008 | 93,190 | 1,589 | 74.00 | 50.4 | Retrospective cohort | EIA | High |
| Mizui et al. 2013 | Japan | 1 | 2,010 | 2,011 | 3,212 | 29 | 66.70 | 56.2 | Retrospective cohort | "Toxin test" | High |
| Barletta et al. 2014 | United States | 1 | 2,001 | 2,008 | 408 | 204 | 69.00 | 56.0 | Retrospective case-control | Not stated in text | Moderate |
| Brown et al. 2014 | Canada | 1 | 2,010 | 2,012 | 2,067 | 127 | 68.00 | 51.0 | Prospective cohort | NAAT / PCR | Moderate |
| Pakyz et al. 2014 | United States | 64 | 2,009 | 2,009 | 14,134 | 5,697 |  | 52.6 | Retrospective case-control | ICD-9 | Moderate |
| Jou et al. 2015 | United States | 1 | 2,011 | 2,011 | 468 | 75 | 59.00 | 52.0 | Retrospective cohort | EIA | Moderate |
| Tabak et al. 2015 | United States | 6 | 2,007 | 2,008 | 78,080 | 323 |  | 45.7 | Retrospective cohort | EIA | Moderate |
| Tartof et al. 2015 | United States | 14 | 2,011 | 2,012 | 401,234 | 2,638 | 60.00 | 39.8 | Retrospective cohort | NAAT / PCR | Low |
| Ticinesi et al. 2015 | Italy | 1 | 2,013 | 2,013 | 633 | 43 | 81.00 | 47.1 | Retrospective cohort | Not specified in text | Moderate |
| Davis et al. 2016 | United States | 1 | 2,009 | 2,014 | 140 | 46 | 71.00 |  | Retrospective cohort | EIA | High |
| Faleck et al. 2016 | United States | 3 | 2,010 | 2,013 | 39,535 | 271 | 67.00 | 56.0 | Retrospective cohort | NAAT / PCR | Low |
| Freedberg et al. 2016 | United States | 4 | 2,010 | 2,015 | 100,615 | 576 |  | 49.2 | Retrospective cohort | NAAT / PCR | Low |
| Press et al. 2016 | United States | 1 | 2,013 | 2,013 | 80,324 | 182 |  | 62.0 | Retrospective cohort | NAAT / PCR | Moderate |
| Forster et al. 2017 | Canada | 3 | 2,004 | 2,014 | 208,104 | 792 | 55.00 |  | Prospective cohort | EIA | Low |
| Na'amnih et al. 2017 | Israel | 1 | 2,007 | 2,014 | 772 | 140 | 70.80 | 37.1 | Retrospective case-control | EIA (Start - 2011), GDH + immunochromatographic test -> PCR (2012 - end) | Moderate |
| Davis et al. 2018 | United States | 5 | 2,014 | 2,016 | 97,130 | 1,481 | 55.00 | 36.7 | Retrospective cross-sectional | NAAT / PCR | Moderate |
| Oh et al. 2018 | United States | 2 | 2,010 | 2,016 | 374,008 | 2,693 | 58.00 | 47.0 | Retrospective cohort | NAAT / PCR, EIA | High |
| Tilton et al. 2019 | United States | 14 | 2,015 | 2,017 | 200 | 100 | 65.75 | 45.0 | Retrospective case-control | NAAT / PCR | High |
| Hung et al. 2021 | Taiwan | 1 | 2,011 | 2,013 | 476 | 28 | 73.80 | 45.5 | Prospective cohort | Anaerobic stool culture | Moderate |
| Root et al. 2021 | United States | 1 | 2,015 | 2,016 | 17,285 | 251 |  | 54.7 | Retrospective cohort | Not specified | High |
| Tilton et al. 2021 | United States | 2 | 2,016 | 2,018 | 362 | 161 | 61.61 | 47.2 | Retrospective case-control | NAAT / PCR | High |
| Vader et al. 2021 | United States | 1 | 2,014 | 2,018 | 682 | 170 | 59.54 |  | Retrospective case-control | EIA | Moderate |
| MacKenzie et al. 2023 | United States | 1 | 2,015 | 2,019 | 18,883 | 99 | 62.00 | 53.2 | Retrospective cohort | NAAT / PCR | Moderate |

**Table S2**: Meta-analyzed effect measures for each exposure on HA-CDI with 95% CI and number of point estimates. An asterisk (*) indicates that the exposure was a combination of other exposures within the group.

| **Factor** | **Odds Ratio** | **Lower Bound** | **Upper Bound** |
| --- | --- | --- | --- |
| **Age** |  |  |  |
| Age (> 65) | 1.90 | 0.93 | 3.90 |
| Age (additional year) | 1.01 | 1.00 | 1.01 |
| **Antibiotics** |  |  |  |
| Antibiotic use | 2.97 | 2.37 | 3.72 |
| Cephalosporins | 2.83 | 0.38 | 21.21 |
| Clindamycin | 1.87 | 0.78 | 4.48 |
| Metronidazole | 0.87 | 0.54 | 1.40 |
| Solid tumor malignancy | 2.16 | 1.08 | 4.33 |
| Charlson comorbidity (per unit) | 1.04 | 0.92 | 1.16 |
| Congestive heart failure | 1.51 | 1.05 | 2.16 |
| Dialysis | 1.81 | 1.13 | 2.92 |
| Female | 1.13 | 0.89 | 1.43 |
| **Gastric acid suppression** |  |  |  |
| Gastric acid suppressant*  H2RA | 1.81  1.69 | 1.47  1.27 | 2.23  2.23 |
| PPI | 1.50 | 1.07 | 2.09 |
| Hypoalbuminemia | 2.00 | 1.28 | 3.11 |
| Mechanical ventilation | 2.38 | 1.34 | 4.23 |
| **Race** |  |  |  |
| Black race | 0.79 | 0.67 | 0.92 |
| Other race | 0.69 | 0.48 | 1.00 |
| **Recent hospitalization** |  |  |  |
| Hospital admission (last 2-3 months)* | 2.11 | 1.81 | 2.46 |
| Hospital admission (last 2 months) | 2.11 | 1.78 | 2.50 |
| Hospital admission (last 3 months) | 2.23 | 1.74 | 2.87 |
| Renal failure | 2.66 | 1.15 | 6.14 |
| Transferred from skilled nursing facility | 2.08 | 1.60 | 2.69 |
| **Ward type** |  |  |  |
| ICU | 3.09 | 0.57 | 16.69 |
| Surgical | 0.87 | 0.60 | 1.27 |

**Table S3.** *A priori* definitions used in QUIPS risk of bias tool

| **Domain** | **Issue** | **Definitions** |
| --- | --- | --- |
| Study Participation | The source population or population of interest is adequately described for: | Hospitalization, timing of CDI |
| Study Participation | The baseline study sample (i.e., individuals entering the study) is adequately described for: | Age, comorbidities |
| Study Attrition | Participants lost to follow-up are adequately described for: | Age, comorbidities |
| Study Attrition | There are no important differences between (definitions) and outcomes in participants who completed the study and those who did not. | Age, comorbidities |
| Study Confounding | All important confounders, including treatments (definitions), are measured. | Age, antibiotics, comorbidities, length of stay |

**Table S4.** Assessing study and event definitions.

| **Title** | **Study sample is clearly identified** | **CDI event is clearly identified** | **Event definition** |
| --- | --- | --- | --- |
| Brown et al. 1990 | Yes | Yes | 1st positive test |
| McFarland et al. 1990 | Yes | Yes | ≥3 BMs for ≥2d without other cause and positive toxin assay after onset |
| Nelson et al. 1994 | Yes | Yes | Positive cytotoxin and history of diarrhea or incontinence ≤4d prior to positive test |
| Yip et al. 2001 | Yes | Yes | Symptoms for ≥2 days ≥72h after admission |
| McCusker et al. 2003 | Yes | Yes | New onset diarrhea and positive toxin A assay and no history of CDAD |
| Dial et al. 2004 | Yes | Yes | >2 instances of watery stool & a positive assay |
| Dubberke et al. 2007 | Yes | Yes | Unformed stool ≥48h post-admission |
| Aseeri et al. 2008 | Yes | Yes | CDI toxin positive by EIA |
| Baxter et al. 2008 | Yes | Yes | A or B toxin on EIA, 3+ days after admission |
| Garey et al. 2008 | Yes | Yes | C. difficile infection confirmed by tissue culture cell cytotoxicity assay after 48hr admission |
| Eddi et al. 2010 | Yes | No | + EIA, but no details about timing |
| Howell et al. 2010 | Yes | Yes | Positive CDI ≥3 days after admission |
| Loo et al. 2011 | Yes | Yes | Diarrhea with cytotoxin assay or toxigenic culture, diarrhea without alternative explanation and pseudomembranes, or pathological diagnosis of CDI. Diarrhea was defined as 3 or more loose stools/day |
| Monge et al. 2011 | Yes | Yes | ≥3 BMs for ≥2d, pseudomembranous colitis or toxin megacolon & C. diff toxin positive |
| Mora et al. 2011 | Yes | Yes | Diagnosed with CDI and diarrhea with toxin assay positive |
| Zilberberg et al. 2011 | Yes | Yes | Positive toxin assay ≥48h after admission |
| Motzkus-Feagans et al. 2012 | No | Yes | Any inpatient with ICD-9 008.45 started on metronidazole or vancomycin PO for ≥3 days after ≥5 days of admission |
| Ahyow et al. 2013 | Yes | Yes | 1st diarrheal stool sample testing positive for toxin A and/or B during inpatient admission at least 2 days after admission |
| Mizui et al. 2013 | Yes | Yes | Positive result on toxin test |
| Barletta et al. 2014 | Yes | Yes | ICD-9 code |
| Brown et al. 2014 | Yes | Yes | Positive NAAT/PCR or positive pathology |
| Pakyz et al. 2014 | Yes | Yes | Discharged patient with any ICD-9-CM code for CDI (008.45) who had also received drug treatment for CDI (e.g. metronidazole or oral vancomycin) for at least 3 days |
| Jou et al. 2015 | Yes | Yes | GDH EIA then toxin A/B EIA |
| Tabak et al. 2015 | Yes | Yes | Positive EIA ≥48h after admission |
| Tartof et al. 2015 | Yes | Yes | Positive test after ≥72h admission |
| Ticinesi et al. 2015 | Yes | Yes | Stool sample with confirmation of toxin assay in patient with diarrhea or pseudomembranes on colonoscopy |
| Davis et al. 2016 | No | Yes | EIA positivity |
| Faleck et al. 2016 | Yes | Yes | Unformed stool, PCR positive, appropriate subsequent therapy |
| Freedberg et al. 2016 | Yes | Yes | Toxin B PCR and unformed stool preceding directed CDI treatment |
| Press et al. 2016 | Yes | Yes | CDI after 72h, must have diarrhea |
| Forster et al. 2017 | Yes | Yes | Unformed stool with A or B toxin on EIA |
| Na'amnih et al. 2017 | Yes | Yes | Acute onset of diarrhea (3 or more stools in a day) and a positive test |
| Davis et al. 2018 | Yes | Yes | Liquid stool and positive molecular test |
| Oh et al. 2018 | Yes | Yes | CDI positive ≥72h past admission |
| Tilton et al. 2019 | No | Yes | PCR ≥48 after admission AND signs/symptoms of CDI |
| Hung et al. 2021 | Yes | Yes | 3 or more unformed BMs/d for 2 or more days |
| Root et al. 2021 | No | No | CDI diagnosed after 72h of admission |
| Tilton et al. 2021 | No | Yes | Positive PCR after day 4 |
| Vader et al. 2021 | Yes | Yes | Symptom onset ≥72h after admission |
| MacKenzie et al. 2023 | Yes | Yes | Subject received antibiotics and antibiotics were indicated for CDI treatment (vancomycin PO/PR or fidaxomicin PO) |
