# Supplementary material for: Meta-analysis of predictors of healthcare-associated Clostridioides difficile infection
Source: Antimicrob Steward Healthc Epidemiol. 2024 Nov 14;4(1):e202. doi: 10.1017/ash.2024.413 (PMC11574607; doi:10.1017/ash.2024.413)
Supplement: Fajnzylber et al. supplementary material 8 — Fajnzylber et al. supplementary material [file S2732494X24004133sup008.docx]

**Supplemental Figures:**

**Figure S1.** Traffic light plot for six domains of QUIPS risk of bias.

**Figure S2.** Forest plot for a sub analysis of proton pump inhibitors (PPIs) that adjusted for both comorbidities (either individually or through an index measure) and age.

**Figure S3.** Forest plots for recent prior hospitalization

**Figure S4.** Forest plots for demographic information. A) Age > 65, B) Age per unit year, C) Sex (female vs. male).

**Figure S5.** Forest plots for antibiotics. A) Any antibiotics, B) cephalosporins, C) clindamycin, D) metronidazole.

**Figure S6.** Forest plots for comorbidities. A) Solid tumor malignancy, B) congestive heart failure, C) renal failure, and D) Charlson comorbidity index.
